# Nocardia empyema in an immunocompromised host: A case report

**DOI:** 10.1016/j.rmcr.2025.102199

**Published:** 2025-04-01

**Authors:** Carlos Ignacio Rodríguez Reyna, Erick Torres Luna, Ajay Sheshadri, Saadia Faiz, Lara Bashoura, Nathan Box

**Affiliations:** a Tecnologico de Monterrey, Escuela de Medicina y Ciencias de la Salud, Av. Ignacio Morones Prieto 3000, Sertoma, 64710, Monterrey, N.L, Mexico; b The University of Texas MD Anderson Cancer Center, Department of Pulmonary Medicine, 1515 Holcombe Blvd., Houston, Texas, 77030, USA; c McGovern Medical School at University of Texas Health, Divisions of Pulmonary, Critical Care Medicine and Sleep Medicine, 7000 Fannin St, Houston, TX, 77030, USA

## Abstract

Patients with myeloproliferative disorders such as primary myelofibrosis are at an increased risk of opportunistic infections including *Nocardia* pneumonia. *Nocardia* can become disseminated with the central nervous system being the primary site affected although any organ system can be involved. Here we present a case of *Nocardia* empyema, a rare and sparsely documented location of infection, in a post matched unrelated donor hematopoietic cell transplant patient. Further complicating this case was the presence of fungal pneumonia in the right lower lobe that led to delayed diagnosis of *Nocardia* pneumonia and empyema as non-resolving nodular opacities were thought to be worsening fungal pneumonia. This case highlights the difficulties in diagnosing *Nocardia* infections, the limitations of our diagnostic tools, the atypical presentation of empyema in an immunocompromised host, and Hickam's dictum; the idea that a patient can have multiple diagnoses occurring simultaneously.

## 1 Introduction

Primary myelofibrosis (PM), a rare form of chronic myeloproliferative disease, is defined by atypical megakaryocytic hyperplasia associated with bone marrow fibrosis as a result of nonclonal fibroblastic proliferation [1]. The treatment may involve the use of hematopoietic cell transplantation (HCT), but this can lead to an increased risk of infection through acquired immune defects and the use of therapeutic immunosuppression to prevent alloimmune complications, particularly graft-versus-host disease (GVHD) [2]. Pulmonary nocardiosis is an uncommon, potentially life-threatening opportunistic infection that mainly affects patients with deficient T-cell mediated immunity [3]. Herein, we present a case of myelofibrosis complicated with a rare form of disseminated nocardiosis.

## 2 Case presentation

We present a 68-year-old man with PM diagnosed 7 years prior after presenting with progressive fatigue, abdominal pain, intermittent nausea and vomiting, and weight loss for one year. A bone marrow aspiration and biopsy were performed, demonstrating a hypercellular bone marrow with an increased number of megakaryocytes with atypical morphology and diffuse marrow fibrosis. A peripheral blood smear showed leukoerythroblastosis, consistent with PM. Therapeutic options were discussed with the patient, who opted for ruxolitinib instead of HCT.

After five years of ruxolitinib treatment, the patient started requiring monthly red blood cell transfusions, and HCT was recommended. He received conditioning with busulfan, fludarabine and thiotepa with reduced post-transplant cyclophosphamide (PTCy) followed by a matched unrelated donor (MUD) HCT. Post-HCT infection prevention treatment included caspofungin, levofloxacin, valacyclovir, and letermovir. GVHD prophylaxis included tacrolimus and mycophenolate mofetil.

**Fig. 1:**
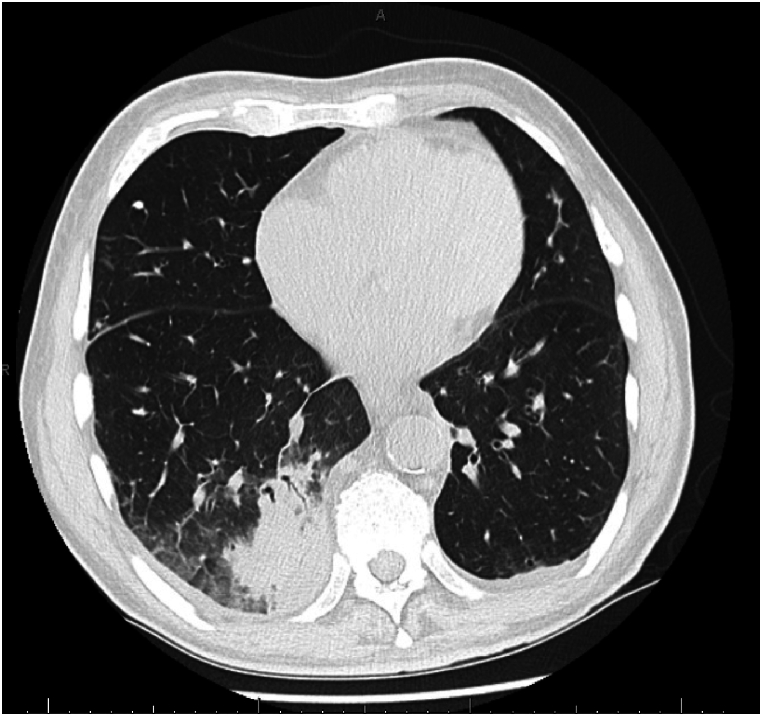
Representative image from CT chest without IV contrast showing right lower lobe consolidation with air bronchograms and surrounding ground glass opacity.

Nine days after HCT patient developed a fever of 101.9F. This was attributed to a transfusion reaction after infusion of packed red blood cells. Thirteen days post-HCT, the patient reported having constant right-sided chest pain at rest. Computed tomography (CT) scan without intravenous contrast of the chest was obtained that revealed the presence of right lower lobe consolidative opacities and scattered bilateral nodular opacities with small pleural effusions consistent with pneumonia (Fig. 1). A bronchoalveolar lavage (BAL) was performed to evaluate for opportunistic pathogens. Culture was negative, but an *Aspergillus* antigen (Ag) index assay was elevated (7.69). In addition, a polymerase chain reaction (PCR) assay for Pneumocystis jirovecii was mildly positive. He was initially started on IV amphotericin B and voriconazole, which was later switched to posaconazole. The *Pneumocystis jirovecii* result was felt to be colonization and not infection. The patient improved and was discharged from the hospital 30 days post-HCT.

Over the next 3 weeks, he developed progressive shortness of breath with exertion but not at rest and a dry cough. He was evaluated by the pulmonary service 53 days post-HCT and found to have an enlarging multiloculated right pleural effusion. A therapeutic and diagnostic thoracentesis was performed, 1650 ml of serosanguinous pleural fluid was evacuated. The results of diagnostic studies are shown in Table 1 and revealed an exudative effusion based on the total protein and lactate dehydrogenase (LDH) levels. Bacterial, fungal, and AFB cultures were negative, and cytology did not show evidence of malignancy.

**Table 1:** Results of first two diagnostic thoracentesis.

Variable	Units	Thoracentesis 53 days post SCT	Thoracentesis 66 days post SCT
		Value	Value
WBC count	Cells/μL	287	1267
RBC count	Cells/μL	46,000	11,000
PMN %	%	35	68
Lymphocytes %	%	17	6
Eosinophils %	%	0	9
LDH	U/L	114	151
Amylase	U/L	11	16
Glucose	mg/dL	114	115
Cholesterol	mg/dL	56	79
Triglyceride	mg/dL	38	57
Protein	g/dL	3.3	4.1

**Fig. 2:**
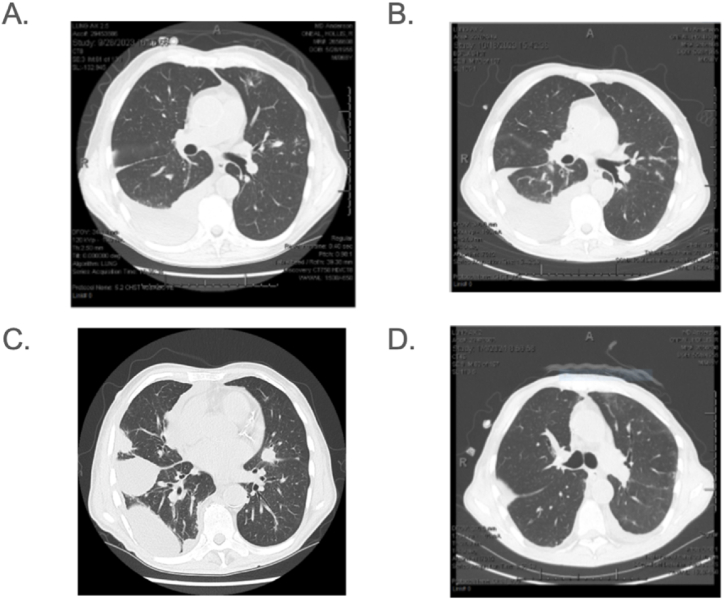
A. CT chest without contrast from 37 days post-transplant and after initial treatment for fungal pneumonia was initiated; B. CT chest without contrast from 65 days post-transplant; C. CT chest without contrast from 80 days post-transplant prior to placement of chest tubes for empyema; D. CT chest without contrast from 143 days post-transplant showing resolution of right sided pleural effusion.

The patient presented 12 days later with complaints of increasing dyspnea, worsening cough, and right-sided chest pain, stating that his symptoms had improved after thoracentesis briefly, but then progressed after a few days. CT of the chest showed parenchymal opacities in the right lung, presumed to be worsening fungal pneumonia, and an increase in a right-sided multiloculated pleural effusion (Fig. 2). The patient was empirically started on cefepime and caspofungin. A second ultrasound-guided right thoracentesis was performed (Table 1), removing 1100 ml of hazy fluid. Pleural fluid pH not obtained but glucose was 115 mg/dL. A chest CT showed new nodular opacities and progression of previous opacities, so a BAL of the right middle lobe lateral segment was performed. Bacterial cultures were found to be negative, and the patient was discharged and returned home on oral posaconazole, daily IV caspofungin via home health infusions, and IV ambisome on a Monday/Wednesday/Friday schedule at an infusion center. Subsequently, a BAL *Aspergillus Ag* index was found to be within normal limits (0.18), and fungal cultures were negative, but the patient did not improve. On day 89 post-HCT, acid-fast bacterial cultures from pleural fluid and BAL showed infection with *Nocardia farcinica,* which was susceptible to trimethoprim/sulfamethoxazole (TMP-SMX), imipenem, moxifloxacin, and Augmentin. On day 92, a third thoracentesis procedure was performed, removing 300 mL of yellow fluid. Following this a right chest 14 French pigtail catheter was placed, and he underwent therapy with intrathoracic tissue plasminogen activator and dornase alfa for empyema. A second catheter was placed for a loculated effusion pocket not drained by first catheter. Both catheters were removed 11 days later. MRI brain performed following discovery of the *Nocardia* infection was negative for CNS involvement. He was started on IV TMP-SMX and intravenous imipenem but developed nausea and vomiting; TMP-SMX was discontinued, and amoxicillin-clavulanic acid was added. He completed 5 weeks of this regimen before the imipenem was switched to oral moxifloxacin. He later developed similar gastrointestinal symptoms due to amoxicillin-clavulanic acid, so this was discontinued, and minocycline was started instead. He completed 6 months in total of antibiotic therapy for *Nocardia* with gradual but complete improvement in symptoms. He had serial CT chests throughout the next year that showed gradual resolution of his pleural effusions and bilateral infiltrates (see Fig. 2).

## 3 Discussion

*Nocardia*, an aerobic actinomycete that appears as a gram-positive branching filamentous bacillus under direct microscopy, represents a significant healthcare challenge due to its diverse clinical manifestations and the risk for disseminated infections in immunocompromised individuals [4]. It is a rare infection with only 500 to 1000 estimated cases in the US each year [5]. It can cause cutaneous only, pulmonary only, or disseminated disease [4]. Diagnosis relies primarily on microbiological culture and imaging studies, particularly in obtaining adequate samples for accurate identification and susceptibility testing. Treatment strategies involve a combination of antibiotics, with TMP-SMX being the mainstay while carbapenems, quinolones, amoxicillin-clavulanic acid, or linezolid may be considered, especially in multi-drug-resistant strains, cases with CNS involvement, or in individuals that progress despite first-line therapy [6]. Treatment duration is typically 6–12 months [5].

This case is unique due to the presence of nocardia empyema, an even rarer form of disease. Review of the literature shows only sparse case reports detailing nocardia empyema. Likely leading to the development of the empyema was the delayed diagnosis of *Nocardia* pneumonia. The diagnosis of *Nocardia* pneumonia is difficult as the CT scan findings are non-specific, staining sensitivity is modest, and cultures can take time to result. Prior studies have shown Gram stain of expectorated sputum had a sensitivity of 78 % [7]. *Nocardia* tends to grow on routine growth media and can result within 2–7 days, however some species grow more slowly and can take up to several weeks to result [7]. Polymerase chain reaction (PCR) has been proposed as another diagnostic tool for diagnosis. A study in 2018 across three French centers showed a sensitivity of 88 % and specificity of 74 % [8]. Unfortunately, *Nocardia* PCR was not utilized in this case. Also confounding his clinical course was the presence of concurrent *Aspergillus* pneumonia. Careful review of his CT scans show that the initial right lower lobe consolidation resolved after only one month of anti-fungal therapy while the numerous bilateral nodular opacities remained and slowly increased in size despite escalating anti-fungal therapy. This along with his *Aspergillus* antigen on repeat BAL resulting negative after previously being elevated suggests he did have a true fungal pneumonia concurrently with *Nocardia*. Due to the fungal pneumonia diagnosis the unresolving pulmonary nodules were attributed to failure of anti-fungal therapy and a second infection was not considered.

The diagnosis of empyema was also delayed despite multiple thoracentesis and pleural fluid analysis. Unlike typical empyema the fluid from the second thoracentesis showed normal glucose and LDH levels less than 3 times the serum level while pH was not checked. Thus empyema was not originally considered in the differential. In addition, the nucleated cell count in the fluid was low from the first two thoracentesis. This is probably due to the immunocompromised state of the patient leading to decreased inflammatory state in the pleural space and therefore relatively low migration of inflammatory cells, low utilization of glucose, and low production of LDH. This highlights the shortcomings of Light's criteria when used in severely immunocompromised patients and its sensitivity in this setting is likely much lower than the >95 % reported in the literature [9]. Review of case reports of *Nocardia* empyema published since 2019 are summarized in Table 2. Unfortunately, most reports did not report the pleural fluid pH or glucose nor even the pleural fluid protein, LDH, or cell counts. This makes it less clear if *Nocardia* infection of the pleural space has a lower tendency to present with the classic purulent fluid that is low in glucose and low pH that typical empyema does or if our patient was unique due to his SCT and immunosuppressed state. Finally the question of whether pleuroscopy could have had a role in this case to diagnose the empyema quicker arises. Like typical empyema our patient was managed with appropriate antimicrobials and drainage of the fluid with chest tubes. Given the rarity of *Nocardia* empyema it is currently unknown if more aggressive source control measures such as surgical decortication are necessary.

**Table 2:** Case reports since 2019 describing cases of *Nocardia* empyema.

Author	Year	Patient Age	Patient Sex	Immunocompromised state	Pleural fluid pH	Pleural fluid glucose	Pleural fluid protein	Pleural fluid LDH	Pleural fluid Cell Count	Nocardia spp.found in pleural culture	Intervention	Outcome
[10]	2019	66	Male	Yes; esophageal cancer and CLL on active chemo	Not reported	Not reported	Not reported (reports exudative effusion)	Not reported (reports exudative effusion)	76,000; 93 % PMNs	N. paucivarans	Chest tube with fibrinolytic therapy	Not reported
[11]	2024	60	Female	history of right lung adenocarcinoma status post neoadjuvent chemo/radiation and pneumonectomy	Not reported	Not reported	Not reported	Not reported	Not reported	N. nova	Pleuroscopy; planned for wash out but expired prior	Expired
[12]	2021	78	Male	Yes, history of bladder & prostate cancer; new diagnosis of Adult T-cell leukemia/lymphoma (ATL) same admission	7.5	7.2	Not reported	1283	2200; 68 % PMNs	N. asiatica	Chest tubes with fibrinolytic therapy	Transferred to other facility for rehab
[13]	2024	81	Male	Relative; history of COPD, active smoker, farmer	Not reported	Not reported	Not reported	Not reported	Not reported	N. otitidiscaviarum	Chest tube placement	Expired
[14]	2025	49	Female	SLE on immunosuppression	Not reported	84	3.4	190	87 % PMNs, no count reported	N. beijingensis	Serial thoracentesis	Not reported
[15]	2022	84	Female	Hepatitis C	Not reported	Not reported	Not reported	Not reported	Not reported	N. farcinica	None	patient had empyema necessitans; died of aspiration event
[16]	2022	55	Male	Renal transplant with immunosuppression	Not reported	Not reported	“Exudative"	“Exudative"	20-30/hpf	N. farcinica	None	Treated successfully
[17]	2024	51	Female	Rheumatoid arthritis, metastatic colon adenocarcinoma on chemotherapy	Not reported	37	3.45	490	820; 79 % PMNs	N. otitidiscaviarum	Chest tube followed by surgical decortication	Treated successfully

Since discharge, the patient slowly returned to his baseline and his current imaging shows minimal residual scar with no new pulmonary impairment despite his delayed diagnosis and adequate treatment. *Nocardia* empyema is a rare presentation of disseminated *Nocardia* infection and requires early recognition, prompt diagnosis, and initiation of appropriate therapy for successful cure. This case highlights the importance of recognizing the limitations of our diagnostic criteria and testing for both *Nocardia* infections and empyema in general. Finally, this case demonstrates that the patient can have multiple diagnoses simultaneously and the importance of re-evaluating the diagnosis if the patient fails to improve with appropriate treatment.

## CRediT authorship contribution statement

**Carlos Ignacio Rodríguez Reyna:** Writing – original draft. **Erick Torres Luna:** Writing – original draft. **Ajay Sheshadri:** Writing – review & editing. **Saadia Faiz:** Writing – review & editing. **Lara Bashoura:** Writing – review & editing. **Nathan Box:** Writing – review & editing.

## Declaration of competing interest

The authors declare that they have no known competing financial interests or personal relationships that could have appeared to influence the work reported in this paper.
